# Erythrocyte selenium concentration predicts intensive care unit and hospital mortality in patients with septic shock: a prospective observational study

**DOI:** 10.1186/cc13860

**Published:** 2014-05-07

**Authors:** Nara Aline Costa, Ana Lúcia Gut, José Alexandre Coelho Pimentel, Silvia Maria Franciscato Cozzolino, Paula Schmidt Azevedo, Ana Angélica Henrique Fernandes, Bertha Furlan Polegato, Suzana Erico Tanni, Rafael Dezen Gaiolla, Leonardo Antonio Mamede Zornoff, Sergio Alberto Rupp de Paiva, Marcos Ferreira Minicucci

**Affiliations:** 1 Department of Internal Medicine, Botucatu Medical School, UNESP - Universidade Estadual Paulista, Av. Prof. Montenegro s/n, Botucatu, São Paulo, SP 18618970, Brazil; 2 Department of Food and Experimental Nutrition, Faculty of Pharmaceutical Science, University of São Paulo, Av. Prof. Almeida Prado, 1280, São Paulo, SP 05508-070, Brazil; 3 Chemistry and Biochemistry Department, Institute of Biological Sciences, UNESP - Universidade Estadual Paulista, Av. Prof. Montenegro s/n, Botucatu, São Paulo, SP 18618970, Brazil

## Abstract

**Introduction:**

Selenoenzymes can modulate the extent of oxidative stress, which is recognized as a key feature of septic shock. The pathophysiologic role of erythrocyte selenium concentration in patients with septic shock remains unknown. Therefore, the objective of this study was to evaluate the association of erythrocyte selenium concentration with glutathione peroxidase (GPx1) activity, GPx1 polymorphisms and with ICU and hospital mortality in septic shock patients.

**Methods:**

This prospective study included all patients older than 18 years with septic shock on admission or during their ICU stay, admitted to one of the three ICUs of our institution, from January to August 2012. At the time of the patients’ enrollment, demographic information was recorded. Blood samples were taken within the first 72 hours of the patients’ admission or within 72 hours of the septic shock diagnosis for determination of selenium status, protein carbonyl concentration, GPx1 activity and GPx1 Pro198Leu polymorphism (rs 1050450) genotyping.

**Results:**

A total of 110 consecutive patients were evaluated. The mean age was 57.6 ± 15.9 years, 63.6% were male. Regarding selenium status, only erythrocyte selenium concentration was lower in patients who died in the ICU. The frequencies for GPx1 Pro198Leu polymorphism were 55%, 38% and 7% for Pro/Pro, Pro/Leu and Leu/Leu, respectively. In the logistic regression models, erythrocyte selenium concentration was associated with ICU and hospital mortality in patients with septic shock even after adjustment for protein carbonyl concentration and acute physiology and chronic health evaluation II score (APACHE II) or sequential organ failure assessment (SOFA).

**Conclusions:**

Erythrocyte selenium concentration was a predictor of ICU and hospital mortality in patients with septic shock. However, this effect was not due to GPx1 activity or Pro198Leu polymorphism.

## Introduction

Overall cellular oxidative burden is regulated by a balance between the rates of reactive oxygen species (ROS) generation and a variety of antioxidant enzymes/pathways. ROS are generally believed to be harmful because they cause oxidative damage to DNA, protein, lipids and other macromolecules. However, at nanomolar concentrations, these free radicals may play an important role in physiological processes, for example, by functioning as a second messenger in signal transduction pathways. Thus, ROS can be deleterious through modulation of signaling networks [1,2]. In addition, oxidative stress and mitochondrial dysfunction have been recognized as key features of severe sepsis and septic shock [3,4]. For this reason, in the last decade, antioxidant supplementation in critically ill patients has been evaluated [5,6]. Heyland *et al*. showed in a meta-analysis that antioxidants, particularly high-dose intravenous selenite, as monotherapy or in combination with other antioxidants, are a safe strategy and are associated with a trend toward reduction in outcomes, including mortality [5].

Selenium is an essential trace element, and its incorporation into selenoproteins is associated with biological functions. For instance, the increased glutathione peroxidase (GPx) activity is known to increase antioxidant defenses and reduce oxidative damage [7]. The finding of low plasma or whole blood selenium concentrations in critically ill patients has encouraged several selenium supplementation trials. Despite promising results, the optimal dose, method and time of administration remain to be established [8-11]. It is probable that the nutritional selenium status previous to intensive care unit (ICU) admission and specific patient characteristics could influence the response to selenium supplementation.

Usually, nutritional status of selenium is commonly assessed by direct measurement of plasma selenium concentration or indirectly by measuring the activity of the GPx [12]. However, studies that evaluated patients with systemic inflammatory response, plasma selenium concentration showed controversial results [13-15]. Heyland *et al*. in the REDOX study showed that baseline median plasma selenium levels were within normal limits in patients with multiple organ dysfunction [15]. In a study involving 91 critically ill patients, Stefanowicz *et al*. recently showed that plasma selenium is affected by the inflammatory response while erythrocyte selenium concentration is unaffected and can be used to reliably assess selenium status across a wide range of selenium intakes [12]. In addition, erythrocyte selenium concentrations reflect longer term nutritional status due to its incorporation in erythrocyte synthesis [16].

Eight GPx enzymes are expressed in humans, and several studies have demonstrated their association with health. Some GPxs (GPx1, GPx2, GPx3 and GPx4) are selenium-containing enzymes and, therefore, are implicated in different typical functions of selenoproteins, including inflammation, angiogenesis, and endothelial function [17]. However, the most studied action of GPxs is related to oxidative stress. Indeed, to protect against oxidative stress, GPxs work in a coordinated manner with other enzymatic antioxidants (superoxide dismutase, and catalase) to convert ROS to H_2_O.

GPx1 is a selenoprotein that consists of four 22 kDa subunits, each of which contains one atom of selenium. Some polymorphisms have been found in human GPx1, and Pro198Leu polymorphism is thought to be functional [18]. Despite the conflicting results, individuals carrying the variant alleles seem to have an altered response to selenium supplementation [19].

Therefore, the objective of this study was to evaluate the association of erythrocyte selenium concentration with GPx1 activity, Pro198Leu polymorphism and with both ICU and hospital mortality in septic shock patients.

## Materials and methods

All procedures were approved by the Ethics Committee of Botucatu Medical School (4063/2011), and have therefore been performed in accordance with the ethical standards laid down in the 1964 Declaration of Helsinki and its later amendments. Written informed consent was obtained from all patients prior to their inclusion in the study. All patients older than 18 years with septic shock on admission or septic shock during their ICU stay who were admitted to one of the three 28-bed ICUs at our hospital between January and August 2012 were prospectively evaluated.

At the time of the patients’ enrollment, demographic information, the acute physiology and chronic health evaluation (APACHE II) score and the sequential organ failure assessment (SOFA) score were recorded. Blood samples were taken within the first 72 hours of the patients’ admission or within 72 hours after septic shock diagnosis for determination of selenium status, protein carbonyl concentration, GPx1 activity and Pro198Leu polymorphism genotyping.

Septic shock was defined as an infection-induced systemic inflammatory response with systolic blood pressure lower than 90 mmHg or a mean arterial pressure lower than 70 mmHg requiring the introduction of vasopressor drugs [20]. All patients were followed during their ICU and hospital stay. The ICU and hospital mortality were recorded.

### Laboratory analysis

Total serum levels of sodium, potassium, magnesium, total calcium, phosphorus, C-reactive protein (CRP), albumin, creatinine and urea were measured using the dry chemistry method (Ortho Clinical Diagnostics VITROS 950™; Johnson & Johnson, New Brunswick, NJ, USA), and lactate was measured using Roche OMNI S™ Blood Gas Analyzer (Roche Diagnostics, Basel, Switzerland). Hemograms were performed with a Coulter STKS hematology autoanalyzer (Coulter Electronics Ltd., Luton, Bedfordshire, UK).

### Protein carbonyl determination

Measurement of protein carbonyl groups is the most widely utilized measure of protein oxidation [21]. Therefore, protein carbonyl levels were analyzed based on the reaction with dinitrophenylhydrazine (DNPH) and the formation of a Schiff base according to the method described by Reznick and Packer [22].

### Selenium status and GPx1 activity

Erythrocyte and plasma selenium concentrations were measured using hydride generation flame atomic absorption spectrometry as previously described [19,23]. The references for plasma selenium concentration were values between 1.07 to 1.27 μmol/L (84 to 100 μg/L), and those for erythrocyte selenium concentration were values between 0.76 to 1.52 μmol/L (60 to 120 μg/L) [24,25].

The glutathione peroxidase activity of red blood cell hemolysate was assessed using the method described by Paglia and Valentine using the Ransel kit (Randox Laboratories Ltd, Crumlin, County Antrim, UK) [26].

### Pro198Leu polymorphism

The DNA was isolated from frozen blood samples with the illustra blood genomicPrep Mini Spin Kit (GE Healthcare, Waukesha, WI, USA). Polymerase chain reaction (PCR) primers based on the human GPx1 gene sequence flanking the 198 polymorphism (rs 1050450) (GPx1 forward primer, 50-TGCCCCTACGCAGGTACA-30; GPx1 reverse primer, 50-TCCCAAATGACAATGACACAG-30) were used to generate a 337-bp amplification product [24]. PCR was performed following a previously described method [27]. The analyses were performed in duplicate.

### Statistical analysis

Data are expressed as the mean ± standard deviation (SD) or the median (including the lower and upper quartiles). Comparisons between two groups for continuous variables were performed using Student’s *t* test and between three groups, one-way ANOVA for parameters with normal distribution. Comparisons between two groups for continuous variables were performed using Mann-Whitney test and between three groups, Kruskal-Wallis followed by Dunn’s *post hoc* test for parameters with non-normal distribution. Fisher’s test or the χ^2^ test was used for all categorical data. Spearman correlation was used to evaluate the association of continuous variables. Logistic regression models were used to predict both ICU and hospital mortality in patients with septic shock. The primary outcome was ICU mortality, and the secondary outcome was hospital mortality. Erythrocyte selenium concentration was tested as a continuous independent variable. Parameters that exhibited significant difference in the univariate analysis were included as independent factors in logistic regression models. The only exceptions were variables with high collinearity among them (urea, albumin, and lactate). Data analysis was performed using SigmaPlot software for Windows v12.0 (Systat Software Inc., San Jose, CA, USA). *P* values lower than 0.05 were considered statistically significant.

## Results

One hundred and ten consecutive patients were evaluated. The mean age was 57.6 ± 15.9 years, 63.6% were male, the median ICU and hospital stays were 7 (3 to 13) and 13 (4 to 22) days, respectively. The mortality rate during the ICU stay was 54.5%, and 63.6% died during hospital stay. The demographic, clinical and laboratory data are presented in Table 1. Patients who died during their ICU stay exhibit higher serum lactate, urea and protein carbonyl concentrations, as well as higher APACHE II and SOFA scores. These patients also exhibited lower serum albumin levels.

**Table 1:** Demographic, clinical and laboratory data of 110 patients with septic shock

**Variable**	**ICU mortality**		***P *** **value**
	**Yes (n = 60)**	**No (n = 50)**	
Age, (years)	58.8 ± 15.7	56.2 ± 16.2	0.395
Male, number (%)	36 (60%)	34 (68%)	0.503
SOFA score	10.0 (7.3 - 12.0)	9.0 (7.0 - 10.0)	0.032
APACHE II score	21.9 ± 7.4	17.7 ± 6.7	0.003
Sepsis focus, number (%)			
Respiratory	34 (57)	27 (54)	0.193
Soft tissue	8 (13)	2 (4)	
Urinary	4 (7)	7 (14)	
Abdominal	12 (20)	9 (18)	
Others	2 (3)	5 (10)	
MV, number (%)	58 (97)	38 (76)	0.003
RBC transfusion number (%)	37 (62)	28 (56)	0.684
Lactate, (mmol/L)	2.2 (1.3 - 3.8)	1.6 (1.1 - 2.2)	0.007
Hemoglobin, (g/dl)	10.9 ± 2.3	11.2 ± 2.4	0.534
Hematocrit, (%)	32.8 ± 6.7	33.8 ± 7.2	0.429
Leucocytes, (10^3^/mm^3^)	16.1 (10.6 - 21.1)	12.7 (9.2 - 17.5)	0.070
Sodium, (mmol/L)	142 (137 - 147)	140 (137 - 146)	0.286
Potassium, (mmol/L)	4.2 (3.8 - 4.7)	4.1 (3.6 - 4.8)	0.631
Phosphorus, (mg/dl)	4.4 (3.4 - 5.4)	3.7 (2.8 - 4.6)	0.073
Total calcium, (mg/dl)	7.6 (7.0 - 8.3)	8.0 (7.5 - 8.3)	0.115
Magnesium, (mg/dl)	2.1 (1.8 - 2.3)	1.9 (1.7 - 2.2)	0.180
Glycemia, (mg/dl)	180 (121 - 247)	177 (129 - 248)	0.977
CRP, (mg/dl)	23.0 (8.0 - 31.5)	23.0 (6.5 - 36.0)	0.511
Albumin, (g/dl)	2.0 (1.7 - 2.5)	2.6 (2.1 - 3.0)	0.002
Urea, (mg/dl)	96.5 (52.3 - 138.5)	54.5 (42.0 - 92.5)	0.004
Creatinine, (mg/dl)	1.3 (0.7 - 2.5)	1.1 (0.6 - 2.5)	0.418
Erythrocyte Se, (μmol/L)	0.38 ± 0.15	0.46 ± 0.14	0.005
Plasma Se, (μmol/L)	0.30 ± 0.12	0.29 ± 0.11	0.787
Protein carbonyl, (nmol/ml)	14.6 (5.2 - 26.1)	5.4 (3.4 - 21.0)	0.021
GPx1, (U/g Hb)	30.1 (20.8 - 37.1)	31.2 (26.1 - 39.0)	0.301

Data are expressed as the mean ± standard deviation (SD), median (including the lower and upper quartiles) or percentage. ICU, intensive care unit; SOFA, sequential organ failure assessment; APACHE II, acute physiology and chronic health evaluation II; MV, mechanical ventilation; RBC, red blood cell; CRP, C-reactive protein; Se, selenium concentration; GPx1, glutathione peroxidase activity.

In our study, the erythrocyte GPx activity was 30.6 (24.0 to 38.4 U/gHb). About 33.3% of the patients presented lower GPx activity than reference values (27.5 to 73.6 U/gHb). However, GPx activity was not associated with mortality. Regarding selenium status, only erythrocyte selenium concentration was lower in septic patients who died in the ICU (Table 1). Importantly, both survivors and nonsurvivors had markedly low selenium concentration.

Our data showed a correlation between erythrocyte selenium levels and GPx (r = 0.193; *P* = 0.05), at the limit of statistic significance. On the other hand, there is no correlation between GPx and protein carbonyl values (r = -0.175; *P* = 0.09) and erythrocyte selenium levels and protein carbonyl values (r = -0.153; *P* = 0.133).

Genotype analysis was performed in 104 patients due to technical problems with the other samples. The genotype frequencies for GPx1 Pro198Leu polymorphism were 55%, 38% and 7% for Pro/Pro, Pro/Leu and Leu/Leu, respectively. These frequencies are consistent with those expected under the Hardy-Weinberg equilibrium. The erythrocyte selenium concentration, GPx1 activity, protein carbonyl and mortality according to Pro198Leu polymorphism genotype are presented in Table 2. Only GP×1 activity was different between groups and higher in Leu/Leu genotype. In addition, there was no association between GP×1 activity, erythrocyte selenium concentration and protein carbonyl when each genotype group was analyzed separately (Table 3).

**Table 2:** Erythrocyte selenium concentration, GPx1 activity, protein carbonyl and mortality according to Pro198Leu polymorphism genotype

**Variables**	**Pro/Pro (n = 57)**	**Pro/Leu (n = 40)**	**Leu/Leu (n = 7)**	***P *** **value**
Erythrocyte Se, (μmol/L)	0.41 ± 0.15	0.42 ± 0.14	0.46 ± 0.12	0.655
Protein carbonyl, (nmol/ml)	13.5 (4.6 - 24.2)	7.5 (4.4 - 25.7)	5.3 (3.1 - 17.5)	0.403
GPx1, (U/g Hb)	28.5 (19.8 - 36.7)	31.1 (27.7 - 41.0)	35.9 (24.7 - 51.5)	0.045
SOFA score	8.8 ± 2.6	10.4 ± 4.3	9.1 ± 1.9	0.067
ICU mortality, number (%)	27 (47)	26 (65)	4 (57)	0.227
Hospital mortality, number (%)	31 (54)	30 (75)	5 (71)	0.105

Data are expressed as the mean ± standard deviation (SD), median (including the lower and upper quartiles) or percentage. GPx1, glutathione peroxidase activity; Se, selenium concentration; SOFA, sequential organ failure assessment; ICU, intensive care unit.

**Table 3:** Correlation among erythrocyte selenium concentration, GPx1 activity and protein carbonyl according to Pro198Leu polymorphism genotype

	**Pro/Pro**		**Pro/Leu**		**Leu/Leu**	
	**Erythrocyte selenium concentration (μg/L)**					
	**r**	***P***	**r**	***P***	**r**	***P***
GPx1, (U/g Hb)	0.231	0.110	0.145	0.389	0.214	0.602
Protein carbonyl, (nmol/ml)	-0.219	0.121	-0.080	0.650	-0.179	0.660

GPx1*,* glutathione peroxidase activity.

In the logistic regression models, the erythrocyte selenium concentration was associated with ICU and hospital mortality in patients with septic shock even after adjustment for protein carbonyl concentrations, SOFA or APACHE II scores (Table 4).

**Table 4:** Logistic regression models for ICU and hospital mortality in 110 patients with septic shock

	**ICU mortality**			**Hospital mortality**		
	**Odds ratio**	**95% CI**	***P *** **value**	**Odds ratio**	**95% CI**	***P *** **value**
Erythrocyte Se	0.950	0.916 - 986	0.007	0.955	0.920 - 0.992	0.017
Erythrocyte Se*	0.914	0.869 - 0.962	<0.001	0.927	0.883 - 0.973	0.002
Erythrocyte Se**	0.945	0.907 - 0.983	0.006	0.950	0.912 - 0.989	0.013

*Adjusted by protein carbonyl concentration, and acute physiology and chronic health evaluation II (APACHE II); ^**^adjusted by protein carbonyl concentration and sequential organ failure assessment (SOFA). ICU, intensive care unit; Se*,* selenium concentration.

## Discussion

The aim of this study was to evaluate the association of erythrocyte selenium concentration with GPx1 activity, Pro198Leu polymorphism and ICU and hospital mortality in septic shock patients. Erythrocyte selenium concentration was a predictor of mortality in these patients. However, this effect was not due to GPx1 activity or Pro198Leu polymorphism.

Septic shock is the major cause of death in the ICU, and despite increased knowledge of the pathogenesis of sepsis and the creation of ‘bundles’ of care by the international consortium ‘Surviving Sepsis Campaign’, the septic shock mortality rate remains high [28-30]. Oxidative stress plays an important role in the development of organ dysfunction and multiple organ failure in critically ill patients [4]. In our study, patients who died during the ICU stay exhibit higher protein carbonyl concentrations, a biomarker of protein oxidative damage [21]. These data reinforce the importance of oxidative stress in septic patients. For this reason, in the last decade, trials with trace elements and vitamin supplementation for critically ill patients have been performed. Among these micronutrients, selenium is particularly important, and despite promising results, further studies are needed to determine the optimal dose and best method and time of administration [5,6,31]. We believed that nutritional selenium status previous to ICU admission and GP×1 Pro198Leu polymorphism could influence the response of selenium supplementation.

Selenium is an essential micronutrient that functions as a component of many selenoproteins in antioxidant and redox reactions. For instance, mortality in Keshan disease is considered to be related to a selenium deficiency associated with an oxidative aggression due to coxsackie infection leading to lethal cardiomyopathy [32]. Therefore, having a previous selenium deficiency could predispose to death patients suffering from septic shock [33].

More than 25 selenoprotein families have been identified, including glutathione peroxidases, phosphohydroxyl-glutathione peroxidase and thioredoxin reductases [7,34]. It is important to note that in these antioxidant enzymes, there is always one selenium atom, in the form of selenocysteine, at the active site required for its activity [35,36]. In healthy individuals, selenoprotein P is the major selenoprotein in plasma, accounting for 52% of the total plasma selenium. Glutathione peroxidase accounts for another 39%, albumin for 9% and free selenium for less than 1% of total selenium [35].

Selenium status varies by country and corresponds to dietary selenium intake and dietary supplements. For instance, in the USA, 50% of the population takes dietary supplements [37]. Therefore, it is difficult to compare plasmatic selenium levels between different studies. In critically ill patients, it is postulated that plasma selenium could be affected by the inflammatory response. However, in the REDOX trial, patients with sepsis presented the same amount of selenium as controls in the plasma [15]. Importantly, similar to our data, other studies showed that critical patients presented lower plasmatic selenium levels [38-40]. The reasons for these discrepancies remain to be elucidated.

Stefanowicz *et al*. showed recently in a noninflamed population a strong positive correlation between erythrocyte and plasma selenium concentration that was not found in inflamed critically ill patients [12]. In addition, in these patients, plasma selenium was low and erythrocyte selenium concentration was normal. Their results suggest that plasma selenium is affected by the inflammatory response while erythrocyte selenium concentration is unaffected and can be used to reliably assess selenium status [12]. In our study, both survivors and nonsurvivors had low selenium concentration. Importantly, this low selenium concentration is in accord with the observed mortality. However, only erythrocyte selenium concentrations were associated with both ICU and hospital mortality in septic shock patients.

To investigate why erythrocyte selenium concentrations were associated with mortality in our patients, we also evaluated GPx1 activity and its Pro198Leu polymorphism. This polymorphism is associated with a cytidine-to-thymidine substitution in exon 2 of the gene, promoting an amino acid change from proline to leucine at codon 198 [19]. Some studies revealed that the Leu allele is associated with lower responsiveness of the enzyme activity to selenium supplementation [41,42]. However, others did not observe this association [43]. In our study, GPx1 activity was higher in patients carrying the variant allele. However, we did not find any correlation between erythrocyte selenium concentration, GPx1 activity and protein carbonyl in the different genotypes. In addition, the different genotypes did not influence mortality in our patients.

It is important to note that there are other polymorphisms in the GPx gene that could also influence its activity, and these were not evaluated in this research. In addition, the association of thioredoxin reductase and selenoprotein P with erythrocyte selenium concentration was not previously evaluated in septic shock patients.

We should consider the limitations of this study. We only included patients from a single medical center. In addition, our sample size was relatively small. Finally, we did not measure whole blood selenium levels, only plasma and erythrocyte selenium concentration.

## Conclusions

In conclusion, erythrocyte selenium concentration was a predictor of ICU and hospital mortality in patients with septic shock. However, this effect was not due to GPx1 activity or Pro198Leu polymorphism.

## Key messages

• Erythrocyte selenium concentration is a predictor of ICU and hospital mortality in patients with septic shock.

• Erythrocyte selenium concentration effect as a predictor of mortality was not due to influence on GPx1 activity or Pro198Leu polymorphism.

## Abbreviations

APACHE II: acute physiology and chronic health evaluation II score; CRP: C-reactive protein; GPx1: glutathione peroxidase 1; ICU: intensive care unit; PCR: polymerase chain reaction; ROS: reactive oxygen species; SOFA: sequential organ failure assessment.

## Competing interests

The authors declare that they have no competing interests.

## Authors’ contributions

JACP and SMFC carried out the molecular genetic studies and were involved in drafting the manuscript. ALG, PSA, BFP, AAHF and RDG participated in the acquisition and analysis of data, and were involved in drafting the manuscript. NAC participated in the acquisition and analysis of data, carried out the biochemical analysis and helped to draft the manuscript. SET performed the statistical analysis and critically revised the manuscript. LAMZ, SARP and MFM participated in the study design and coordination, helped to draft the manuscript, and critically revised it. All authors read and approved the final manuscript, and agree to be accountable for all aspects of the work.
